# The presence of dominant follicles and corpora lutea does not perturb response to controlled ovarian stimulation in random start protocols

**DOI:** 10.1038/s41598-020-67151-x

**Published:** 2020-06-22

**Authors:** Francesca Filippi, Edgardo Somigliana, Andrea Busnelli, Cristina Guarneri, Stefania Noli, Liliana Restelli, Paolo Vercellini

**Affiliations:** 1 Fondazione IRCCS Ca’ Granda Ospedale Maggiore Policlinico, Milan, Italy; 2 Università degli Studi di Milano, Milan, Italy

**Keywords:** Endocrine reproductive disorders, Chemotherapy

## Abstract

The advent of random start protocols to shorten the time needed to store oocytes in women with malignancies has represented an important improvement in the field of fertility preservation. However, Randomized Controlled Trials are difficult to implement in this area and available evidence that supports this approach remains modest. To shed more light on this issue, we compared the follicular development between the ovary carrying the dominant follicle or the corpus luteum and the contralateral resting ovary in 90 women who underwent random start controlled ovarian stimulation (COS). In fact, ovarian response did not differ between the two ovaries. Subgroup analyses according to the phase of the cycle at the initiation of COS, the type of malignancy, the use of letrozole and the magnitude of the ovarian response did not allow to identify any condition showing a difference in the follicular response between the active and the resting ovaries. In conclusion, follicular growth does not seem to be perturbed by the presence of a dominant follicle or a corpus luteum.

## Introduction

The advent of random start protocols to shorten the time needed to store oocytes in women with malignancies has represented an important improvement in the field of fertility preservation^1,2^. Indeed, women with cancer who are candidate to oocytes cryopreservation can promptly initiate controlled ovarian stimulation (COS), regardless of the cycle phase^3–6^. This approach has allowed to overcome most concerns about the possible detrimental impact of a delay in the initiation of radio or chemotherapy on cancer prognosis^7–9^. Noteworthy, in selected situations, women have even sufficient time to perform two COS cycles in a row, thus increasing the total number of frozen oocytes^10,11^.

The patent clinical advantages of random start protocols have facilitated their rapid spread worldwide. Several case series and comparative studies have been published over the last few years^4,10,12–18^. They are reassuring and generally support the feasibility and effectiveness of these protocols. However, the quality of the available evidence remains modest. To note, there are no randomized controlled trials (RCTs) comparing the effectiveness of random start versus conventional protocols.

In this study, we aimed at providing more evidence on the effectiveness of random start protocols using a different but previously validated approach, i.e. the intra-patient comparison of ovarian responsiveness in the two ovaries of the same patient^19,20^. Specifically, follicular development was compared between the ovary carrying the dominant follicle or the corpus luteum at the beginning of the COS and the contralateral resting ovary. This study design could reveal whether local or paracrine factors associated to the ovulatory activity of the ovary may negatively influence adjacent ovarian responsiveness. Of relevance here is that both dominant follicles and corpora lutea can secrete a plethora of factors with paracrine functions such as in particular the complex network of sex steroids and their metabolites^21^, angiogenic factors^22^ and members of the transforming growth factor-beta (TGF-β) superfamily^23^. In this study, we tested the hypothesis that exposing follicles that are growing under controlled ovarian stimulation to this non-physiological paracrine milieu could perturb their development.

## Materials and methods

Women with malignancies who underwent COS for fertility preservation at the Infertility Unit of our hospital between January 2013 and June 2019 were retrospectively reviewed. The main inclusion criterion was the presence of a unilateral dominant follicle (i.e. mean diameter >11 mm) or a unilateral corpus luteum on day 1 of COS. Exclusion criteria were as follows; (1) previous ovarian surgery (mainly removal of ovarian cysts), (2) presence of ovarian cysts, (3) last menses reported to be less than 5 days earlier, (4) presence of bilateral dominant follicles or bilateral corpora lutea (women with two follicles or two corpora lutea in the same side could conversely be included), (5) cycle cancelled prior to oocytes retrieval. Women were also excluded if they did not have previous sexual intercourses because, in such cases, cycle monitoring was done using transabdominal rather than transvaginal ultrasound (and accuracy of the former was deemed insufficient for the aim of the present study). Women who underwent more than one COS cycle were included only for the first cycle. The experimental protocol was accepted by the local Institutional Review Board (*Comitato Etico Area B – Milano*). Methods were in accordance with local guidelines and regulations. At the time of the studied cycle, all women were also requested to sign a generic informed consent for their data to be used for any retrospective research that could be subsequently designed. Those denying this consent were excluded from the study.

Women were managed as previously reported in details^12,17^. Briefly, once referred by the oncologists to our Unit, patients underwent a transvaginal ultrasound (US) to assess the presence of gynecological disorders and to obtain information regarding ovarian reserve by assessing Antral Follicle Count (AFC). They were then counseled about the pros and cons of oocytes cryopreservation and those accepting to cryopreserve their oocytes initiated gonadotropins the same day. When doubtful, they were offered to refer some days later and, if they accepted to initiate treatments, an US evaluation was repeated prior to start. Ovarian hyperstimulation was obtained using a combination of Corrifollitropin, recombinant FSH and GnRH antagonists. Letrozole tablets 5 mg daily was concomitantly given to women with breast cancers that displayed estrogens or progesterone receptors. It was also given if the definitive histological examination for the presence of these receptors was not available at the time of COS. Ovulation trigger was obtained with the administration of GnRH agonist and oocytes retrieval was performed 36 hours later. According to the policy of our Unit, an in-depth US assessment recording all follicles with a mean diameter ≥11 mm was systematically done on the day of trigger. All follicles were aspirated and, since January 2017, the number of oocytes retrieved was recorded separately for the two ovaries. Clinical information regarding patients’ history, cancer diagnosis, US monitoring and oocytes retrieved was obtained from patients’ charts.

Cycle phase was defined based on US findings. Specifically, women were considered in the follicular phase in the presence of a dominant follicle (mean diameter ≥11 mm) whereas they were considered in the luteal phase in the presence of a corpus luteum as described elsewhere^21^. All ultrasounds were done by two of the authors (F.F and E.S.) with long-lasting experience in gynecological sonography.

Data was analyzed using the Statistical Package for Social Science (SPSS 23.0, IL, USA). The ovary carrying the dominant follicle or the corpus luteum at the beginning of COS was considered *active* whereas the contralateral ovary without these functional findings was considered *resting*. The sample size (about 90 women) was decided *a priori* on the primary aim of the study, i.e. the number of developing follicles per ovary. Specifically, this calculation was done to keep the 95% CI of the estimated proportion of follicles developing in the active ovary of + /− 10% (http://www.openepi.com/SampleSize/SSPropor.htm). With this precision, we could expect to demonstrate a significantly lower response in the active ovaries if the rate of those responding less was above 60%. Indeed, in this situation, the lower limit of the 95% CI of the observed proportion would be above 50%. Data was described as mean ± SD, median (Interquartile range: IQR) or number (%), as appropriate. The 95% Confidence Interval (95% CI) of proportions was calculated using a binomial distribution model. Intrapatient comparisons of the number of developed follicles and oocytes retrieved per ovary were done using the non-parametric Wilcoxon test for paired data. P values below 0.05 were considered statistically significant.

## Results

**Figure 1:**
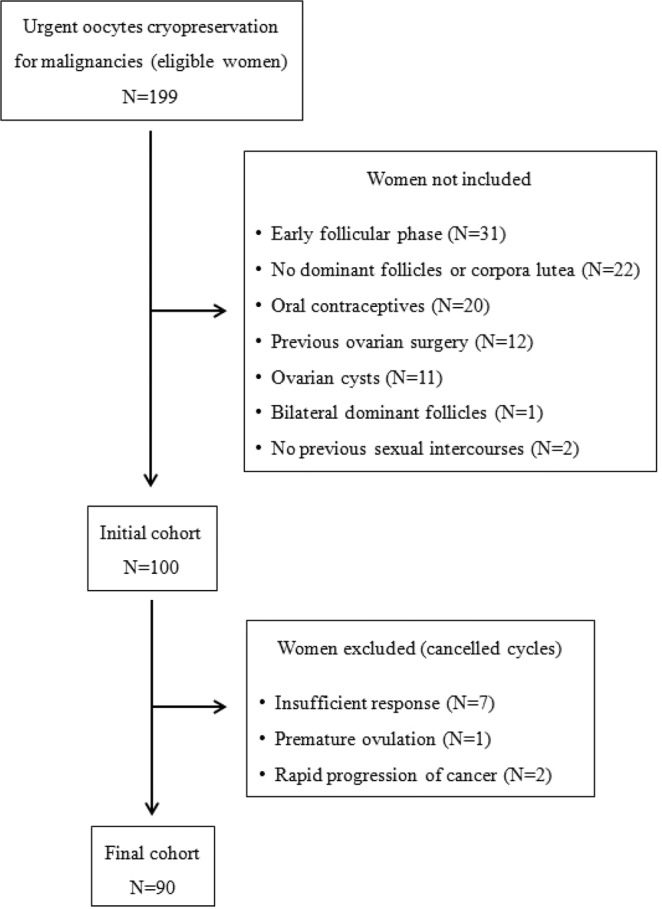
Flow-chart of the selection phase. Overall, more than half of the eligible women could not be informative because they did not fullfill our selection criteria (109 out of 199). The most common reason for exclusion was the referral in the early menstrual cycle phase (N = 31), the absence of dominant follicles or corpora lutea at basal ultrasound (N = 22) and the assumption of oral contraceptives (N = 20).

Ninety women satisfied our selection criteria. The flow-chart of the selection phase is depicted in Fig. 1. Baseline clinical characteristics of the selected women and their cycle outcome are illustrated in Table 1. Twenty-nine (32%) women initiated the stimulation in the proliferative phase (presence of a dominant follicle) whereas the remaining 61 (68%) started in the luteal phase (presence of a corpus luteum). In the former group, the median (IQR) time between the initiation of the last menses and the initiation of COS was 11 (8–15) days. In the latter group, it was 22 (18–27) days (p < 0.001). All the other clinical characteristics (including the duration of stimulation and the total dose of gonadotropins used) did not differ between the two groups (data not shown).

**Table 1:** Baseline characteristics and cycle outcome in the selected women (n = 90).

Characteristics	N. (%) or Mean ± SD or Median (IQR)
Age (years)	31.4 ± 5.8
BMI (Kg/m^2^)	21.9 ± 3.5
Smoking	4 (4%)
Previous deliveries	9 (10%)
Seeking pregnancy at the time of the diagnosis	10 (11%)
Serum AMH (ng/ml)	2.7 (1.5–4.1)
Total AFC	19 (13–26)
**Indication to oocytes cryopreservation**	
Breast cancer	57 (63%)
Lymphoma	20 (22%)
Others	13 (15%)
**Cycle phase at initiation of COS**	
Follicular phase	29 (32%)
Luteal phase	61 (68%)
Time since last menstruation (days)	18 (13–26)
**Drugs used**	
Corrifollitropin 100–150 mcg	79 (88%)
Estimated total dose of recombinant FSH (IU)^a^	2,525 (2,000-3,012)
Duration of stimulation (days)	11.2 ± 1.7
N. of women using letrozole	45 (50%)
N. of developped follicles (diameter ≥11 mm)	18 (12–26)
N. of oocytes retrieved	15 (8–20)
N. of mature oocytes retrieved (frozen)	10 (5–16)

IQR: Interquartile Range. N.: Number

^a^Estimated total dose was calculated adding the total dose of daily gonadotropin administered to 1400 IU for those using also corrifollitropin^12^.

**Figure 2:**
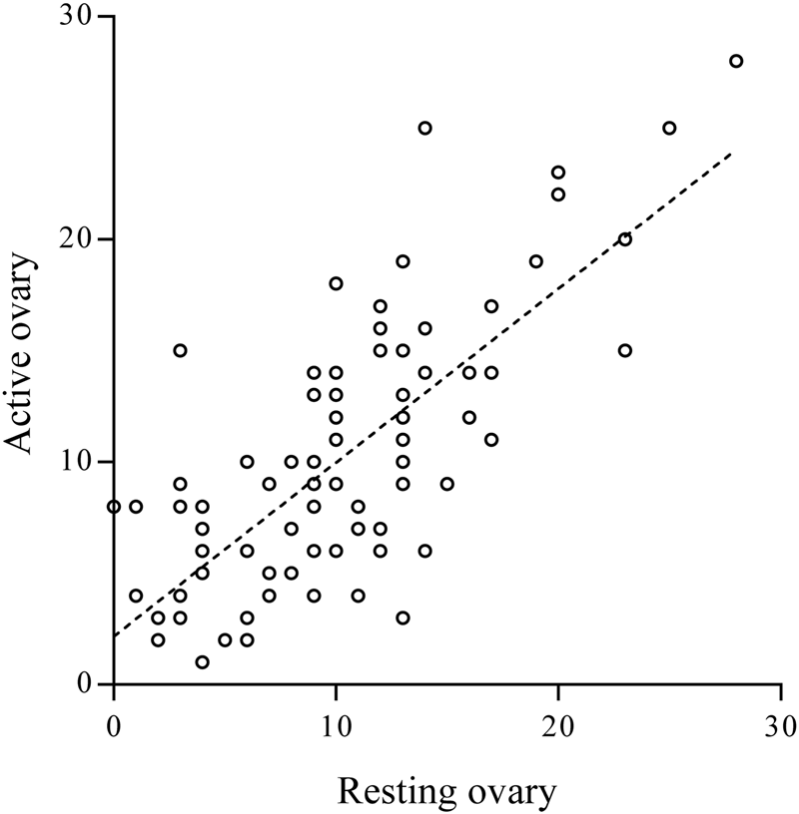
Number of follicles developing in the active (Y axis) and resting (X axis) ovaries. Each woman is represented by a single point. The dotted line represents the linear regression curve (Coefficient of correlation R^2^ = 0.55, p < 0.001).

Thirty women showed a lower response in the active ovary, corresponding to 42% (95% CI: 32–53%). The number (IQR) of growing follicles at the time of the trigger in the active and resting ovaries was 9 (6–14) and 10 (6–13), respectively (p = 0.67). This result is illustrated in Fig. 2. The relative contribution of the active ovary to the total amount of growing follicles was 50% (95% CI: 40–57%). The median (IQR) ratio between growing follicles and basal AFC in the active and resting ovaries was 0.91 (0.65–1.33) and 1.00 (0.70–1.20), respectively (p = 0.09).

In women recruited after January 2017 when collected oocytes were counted separately for the two ovaries (n = 23), the median (IQR) number of oocytes retrieved in the active and resting ovaries was 7 (4–10) and 9 (5–14), respectively (p = 0.38). The median (IQR) ratio between the number of oocytes retrieved and the number of developed follicles was 0.67 (0.57–1.00) and 0.84 (0.67–1.00), respectively (p = 0.24).

Subgroup analyses according to the phase of the cycle at the initiation of ovarian hyperstimulation, the type of malignancies, the use of letrozole and the magnitude of the ovarian response are shown in Table 2. In none of these subgroups, a difference in the follicular response between the active and the resting ovaries emerged. In addition, we performed subgroup analyses for older women (age >35 years, n = 26) and for those with low ovarian reserve (total AFC < 10, n = 6). The number of women with a lower response in the active ovary in these two groups was 13 (50%, 95% CI: 32–68%) and 2 (33%, 95% CI: 10–70%), respectively. The median (IQR) number of developed follicles in the active and resting ovaries was 8 (5–13.25) and 8.5 (4–12.25) for the former comparison (p = 0.67) and 3.5 (2–10.5) and 3 (2.75–6) for the latter (p = 0.50), respectively.

**Table 2:** Number of developing follicles in the active and resting ovaries: subgroup analyses.

Subgroups	N. of women	Cases (%) with a lower response in the active ovary [95% CI]	Developped follicles		
			Active ovary	Resting ovary	p
**Cycle phase**					
Follicular	29	14 (48%) [31–66%]	8 (5–13)	10 (5–13)	0.37
Luteal	61	24 (39%) [28–52%]	9 (6–15)	10 (6–13)	0.88
**Diagnoses**					
Breast cancer	57	23 (40%) [29–53%]	8 (5–14)	10 (5–13)	0.86
Lymphoma	20	8 (40%) [22–61%]	10 (7–14)	10 (8–14)	0.89
Others	13	7 (54%) [29–77%]	9 (4–13)	9 (6–14)	0.62
**Antiestrogens**					
Letrozole	45	14 (31%) [20–46%]	9 (5–15)	9 (4–13)	0.33
None	45	24 (53%) [39–67%]	9 (6–12)	10 (8–13)	0.10
**Ovarian response**					
Total follicles <20	53	26 (49%) [36–62%]	6 (4–8)	7 (4–9)	0.25
Total follicles ≥ 20	37	12 (32%) [20–49%]	14 (12–18)	13 (12–17)	0.49

The 95% CI of the proportion of cases with lower response was calculated using a binomial distribution model

The number of developing follicles is reported as median (interquartile range) and compared using the paired non parametric Wilcoxon test.

## Discussion

Based on our data, ovarian response in the presence of dominant follicles or corpora lutea is not altered. The number of developed follicles in the ovaries that were resting and in those that were active at the beginning of COS was similar. Secondary analyses confirmed the robustness of this result since we failed to identify any subgroup showing impaired responsiveness in the presence of dominant follicles or corpora lutea.

In general, our study supports the validity of random start protocols and results are in line with previous evidence on this subject^4,10,12–18^. However, to the best of our knowledge, our study design has not been previously used to investigate this issue and adds new information. Intra-patient comparisons of ovarian response in the two gonads have been extensively used to evaluate the impact on ovarian reserve of the presence of ovarian cysts (recruiting women with unilateral lesions) or a history of ovarian surgery (recruiting women operated in only one ovary)^19,20^. This study design allowed to overcome several confounders that are generally present and not surmountable in comparative studies. Indeed, both ovaries were exposed to extremely similar conditions and paired analyses could be done, thus increasing the statistical power. In the particular setting of our investigation, this study design allowed to disentangle whether the presence of dominant follicles or corpora lutea could display local detrimental effects on follicular growth. To note, previous studies evaluating ovarian response globally (i.e. evaluating the contribution of both ovaries) could not rule out a local detrimental effect because the resting ovary could compensate for the active one, thus diluting and harboring this potential detrimental effect. Overall, our negative results should be viewed as an additional important evidence supporting the appropriateness of random-start protocols. In addition, our findings can be used to reassure women entering a random start protocol in the presence of a dominant follicle or a corpus luteum. Indeed, the effectiveness of the program cannot be expected to be negatively influenced. This could be particularly important for women who have only one functional ovary because of previous ovarian surgery.

The growth of a dominant follicle and subsequent formation of a corpus luteum causes a profound rearrangement of the ovary, from both a structural, vascular and biochemical point of view. Not only local estrogens, androgens and progesterone markedly rise, but also their metabolites are significantly enhanced^21^. To note, some of these metabolites are biologically active and their functions differ from those of estrogen. Given the capacity of sex steroids to diffuse through tissues, one may reasonably claim some effects on the adjacent follicles that are in earlier phases of development. In addition, it is noteworthy that several proteins with paracrine functions also rise consistently, such as in particular AMH, inhibins, activins, vascular endothelial growth factor (VEGF) and proteins of the TGF-β superfamily^22,23^. The latters include TGF-β, the bone morphogenetic proteins BMP-2, BMP-4, BMP-5, BMP-6, BMP-7 and BMP-15 and the growth and differentiation factor-9 (GDF-9)^22,23^. All these factors are differentially produced by oocytes, follicular and luteinic granulosa cells and theca cells and interact within a complex network that contribute to the regulation of the local vascularization, granulosa cell proliferation, follicle survival and growth, luteinization and atresia^23^. It is well-known that some of these factors may regulate the recruitment of primordial follicular (in particular AMH) but their effects on the development of follicles in their last gonadotropins-dependent phase is difficult to infer based on biological knowledge. For this reason, we hypothesized that some detrimental effects could actually occur. However, the results emerging from our study do not support this concern. They actually tend to rule out any major detrimental effect, at least on the growth of the follicles. The gonadotropins mediated follicular growth actually prevails over possible local perturbing effects.

Some limitations of our study should be acknowledged. Firstly, even if the condition of resting-active ovary may be claimed to randomly occur between the two gonads of the same patient, one cannot exclude that the ovarian reserve may be higher in active ovaries. This could temper the possible detrimental impact of the presence of a dominant follicle or a corpus luteum. However, we do not deem this possible confounder of relevance considering that ovulation rate is reported to be similar in the two ovaries in the general population^24,25^ and that we excluded women with ovarian cysts or with a history of ovarian surgery. In addition, no difference between the two gonads emerged when we compared the ratio between the number of developing follicles and basal AFC.

Secondly, the study is retrospective. A prospective study design would have allowed to retrieve more information. Indeed, even if we deem highly reliable the data on the follicular response (all scans were done by only two physicians with a long-lasting experience in COS and according to the policy of the unit all follicles were measured and recorded on the day of hCG administration), some other information is missing or unreliable. In particular, we lack data on the quality of the folliculogenesis and the competence of the retrieved oocytes. Even if we obtained data on the median number of oocytes retrieved per follicle in a subgroup of women and failed to detect any difference, more data is needed to evaluate the capacity of these gametes to achieve a live birth. In theory, the dominant follicle or the corpus luteum could release some agents (sex steroids but possibly also other factors) that can diffuse through the ovarian stroma and reach the adjacent growing follicles. Even if no effect on responsiveness could be documented, one may hypothesize that this local spread of paracrine factors may somehow disturb the process of folliculogenesis, ultimately affecting the quality of the oocytes and the chances of live birth. To note, evidence on the chances of pregnancy with oocytes obtained within a program of fertility preservation for cancer is still very scanty^26^. Further studies with a prospective recruitment and long term follow-up reporting also on the chances of live birth are required for a definitive answer.

Thirdly, our population was generally young (mean age of 31 years) and the ovarian reserve was fine (median AMH and AFC of 2.7 ng/ml and 19, respectively). Therefore, even if our subgroup analyses based on responsiveness (<vs. ≥20 follicles) did not highlight any effect, we cannot definitely exclude that the presence of dominant follicles or corpora lutea may be specifically detrimental in older women or in women with compromised ovarian reserve. We did subgroup analyses to address this possibility and failed to highlight any impact but the sample size was insufficient for definitive conclusions. More in general, all subgroup analyses in our study were underpowered and results are exposed to a type II error. Larger studies are therefore required for confirmation.

Fourthly, our sample size justification could be viewed as factitious because we aimed at demonstrating a dichotomous effect (a superiority of the resting ovary) and a limited effect (superiority in more than 50% of cases). One could argue that calculating the sample size based on the mean difference in response between the two ovaries would have been more reasonable. However, this approach would have also been factitious and complex because of the non-normality of the distribution of the number of developed follicles and thus the inappropriateness of the use of parametric statistics.

Considering limitations, it has finally to be added that RCTs that obviously represent the best study design to provide robust evidence are extremely difficult to set and run in this context. Indeed, women with cancer may not represent an ideal population for a randomized trial due to time constraints. However, such trial may be considered in non-cancer patients or in those without time urgency.

In conclusion, our study confirms the validity of random start protocols in terms of ovarian response in young women with good ovarian reserve. Further evidence is needed for a definitive and robust conclusion for older women and for those with reduced ovarian reserve. In addition, there is the pressing need to obtain information on the quality of the retrieved oocytes..
